# Valproic acid and bladder healing: an experimental study in rats

**DOI:** 10.1590/0100-6991e-20223399-en

**Published:** 2022-11-10

**Authors:** MARIA DE LOURDES PESSOLE BIONDO-SIMÕES, MOACIR OLIVEIRA DALL’ANTONIA, MATHEUS PRINCE GOEHR, RACHEL BIONDO-SIMÕES, SÉRGIO OSSAMU IOSHII, ROGÉRIO RIBEIRO ROBES

**Affiliations:** 1 - Universidade Federal do Paraná, Técnica Cirúrgica e Cirurgia Experimental - Curitiba - PR - Brasil; 2 - Universidade Federal do Paraná, Programa de Pós-graduação em Clínica Cirúrgica - Departamento de Cirurgia - Curitiba - PR - Brasil; 3 - Universidade Federal do Paraná, Departamento de Patologia da UFPR - Curitiba - PR - Brasil

**Keywords:** Valproic Acid, Wound Healing, Epigenesis, Genetic, Cell Proliferation, Urinary Bladder, Ácido Valproico, Cicatrização, Proliferação de Células, Bexiga Urinária, Epigênese Genética

## Abstract

**Purpose:**

to recognize the effects of valproic acid (VPA), an epigenetic drug, on the bladder healing process, in rats.

**Method:**

twenty male Wistar rats were divided in two groups: experimental (A), treated with VPA (150mg/Kg/day), and control (B) with 0.9% sodium chloridrate. Healing was analyzed on the third and seventh days, evaluating the inflammatory reaction, collagen synthesis and angiogenesis.

**Results:**

inflammatory reaction on the third day was minimal and acute in both groups. On the seventh day, it was subacute in both groups, moderate intensity in group A and minimal in group B (p=0.0476). Collagen III intensity, marked by immunohistochemistry, was similar in both groups. Collagen I intensity on the third day was similar in both groups, but on the seventh day it was higher in experimental than control (p=0.0476). Collagen evaluation by picrosiriusred allowed to verify that the presence of collagen III was similar in both groups (p=0.3312) on the third day, and it was higher in control on the seventh day (p=0.0015). Collagen I showed similarity on the third day (p=0.3100), and it was higher in control on the seventh day (p=0.0015). Vessel marked with anti-SMA counting showed fewer vessels on the third (p=0.0034) and seventh day (p=0.0087) in experimental group. The lower intensity of angiogenesis was confirmed with anti-CD34, on the third day (p=0,0006) and on the seventh day (p=0,0072).

**Conclusion:**

VPA determined alterations in the bladder healing process, in rats, with lower collagen density and less angiogenic activity, but without compromising the integrity of the organ.

## RESUMO

**Objetivo:**

reconhecer os efeitos do ácido valpróico (VPA), uma droga epigenética, no processo de cicatrização da bexiga, em ratos.

**Método:**

vinte ratos Wistar machos foram divididos em dois grupos: experimental (A), utilizando VPA (150mg/Kg/dia), e controle (B), tratados com cloreto de sódio 0,9% por gavagem. A cicatrização da bexiga foi analisada no terceiro e sétimo dia, estudando-se a reação inflamatória, síntese de colágeno, reepitelização e angiogênese.

**Resultados:**

a reação inflamatória no terceiro dia foi mínima e aguda em ambos os grupos. No sétimo dia, foi subaguda em ambos os grupos com intensidade moderada no grupo A e mínima no grupo B (p=0,0476). A intensidade do colágeno III, marcada pela imuno-histoquímica, foi semelhante nos dois grupos, nos dois tempos estudados. A intensidade de colágeno I no terceiro dia foi semelhante nos dois grupos, e maior no sétimo dia no grupo experimental (p=0,0476). A avaliação do colágeno pelo picrosiriusred mostrou que a presença de colágeno III foi semelhante em ambos os grupos (p=0,3312) no terceiro dia, e maior no controle no sétimo dia (p=0,0015). O colágeno I foi semelhante no terceiro dia (p=0,3100), e maior no controle no sétimo dia (p=0,0015). A contagem de vasos marcados pelo anti-SMA mostrou menos vasos no terceiro (p=0,0034) e sétimo dia (p=0,0087) no grupo experimental, confirmado pelo anti-CD34, no terceiro (p=00006) e no sétimo dia (p=0,0072).

**Conclusão:**

o VPA determinou alterações no processo de cicatrização da bexiga, em ratos, com menor densidade de colágeno e menor atividade angiogênica, mas sem comprometer a integridade do órgão.

## INTRODUCTION

The self-regenerative capacity is intrinsic and universal to living beings^1^. As the main structural barrier to environmental aggressors (physical, chemical, or biological) we have the skin, pluristratified squamous keratinized tissue endowed with vast cellular communication among themselves and with the body. The loss of cellular interaction triggers immediate activation of homeostatic molecular events, seeking to restore functional integrity^2^. Thus, healing begins, a complex process that involves migration of inflammatory cells, synthesis of granulation tissue, collagen deposition, and maturation, as well as wound remodeling^3^. 

Recent studies on valproic acid (VPA), a drug used to treat epilepsy and bipolar disorder, have shown it to be responsible for the reduction of some types of cancers and their invasive potential, which is classified as an epigenetic. The epigenetics’ mechanism of action lies in the structural change of DNA, resulting in changes in transcription, translation, and replication of genes. This mechanism includes the alteration of methylation (hypermethylation or hypomethylation) of histones and nonhistone proteins, chromatin remodeling, and changes in gene expression by non-coding RNAs (ncRNAs)^4^. In the case of VPA, it acts as a class I selective inhibitor of histone deacetylases enzymes (HDACs)^5^, responsible for removing methyl grouping from histones, thus leaving the S phase, completing G2 phase and cell division. By inhibiting HDACs, specifically HDAC1, HDAC2, HDAC4 overexpressed in bladder cancer^6^, DNA remains hypermethylated, blocking the cell cycle and preventing disordered mitoses^7^.

Previous studies have shown that VPA interacts in cell migration and cell-binding proteins, but not in cell proliferation, by mechanisms such as protein 53 (p53), decreasing the activity of these cells^8^. In addition, VPA induces thrombocytopenia mainly in women, which may be due to a greater suppression in the formation of platelets in the bone marrow or because it favors the increase of peripheral destruction by anti-platelet antibodies^9^. The VPA also induces a reduction of secretion of vascular endothelium growth factor (VEGF), interfering in angiogenesis, as well as the decrease in the expression and activity of endothelial nitric oxide synthase (eNOS), reducing the formation of new vessels^10^. Fibrinogen also has its levels altered with VPA, by reducing fibrin precursors and increasing fibrinolysis, facilitating the breakdown of fibrin exudate, and thus reducing the cell adhersion^10^.

Cancer treatment using the association of chemotherapy and radiotherapy with epigenetics has been growing for various types of tumors, such as bladder^11^, and prostata^12^. Bladder tumor is the fifth most frequent neoplasm in men and the twelfth in women. In 2012, 165,000 deaths were recorded worldwide due to this cause^13^. In Brazil, the 2018 estimate was 9.480 new cases (6.690 in men and 2.790 in women). In 2017, 4.355 deaths from bladder cancer (3.021 men and 1.933 women) were reported in Brazil^14^.

Tumor patients often receive preoperative radiotherapy and chemotherapy, and it can be associated with an epigenetic in the future, increasing the rate of recovery and decrease of the tumor population’s resistant to certain drugs^15^ ^,^ ^16^. Considering those patients, many of this group will undergo surgical interventions, therefore it is important to study the influence of these medicines on the wound healing process.

The objective of this work was to analyze the action of valproic acid in the bladder healing process in rats.

## METHODS

The experiments were performed in accordance with the Brazilian Guidelines for the Care and Use of Animals for Scientific and Didactic Purposes, edited by the Ministry of Science, Technology, and Innovation, National Council for the Control of Animal Experimentation (CONCEA), in 2013, and the Federal Law No. 11.794, on October 8, 2008. The project was approved by the Ethics Committee on Animal Use of the Sector of Biological Sciences of the Federal University of Paraná (CEUA/BIO - UFPR) on October 22, 2019, receiving the N°. 1.320, process 23075.064161/2019-71. To calculate the sample size, data from previus experiments in this line of research were used for an alpha error of up to 0,05 (5%), sample power of 1- beta error. This calculation was part of the project evaluated by CEUA, with the purpose of respecting the 3 Rs.

Twenty male Wistar rats - Rattus norvegicus Albinus, Rodentia Mammalia -, 120-140 days old and weighing 462.3g ± 33.7g, from the Bioterium of the Federal University of Parana, were randomly allocated into two main groups: experimental (A) and control (B), who received the solutions by oral gavage. Fasting was maintained for 3 hours before the interventions, without water restriction. 

The experimental group was treated with Valproic Acid solution in the doses 150mg/Kg, once a day, started three hours before the procedure, and maintained until the expected date for euthanasia. The control group was treated with 0.9% isotonic saline (NaCl), in the same amount. These animals were subdivided into lots according to the euthanasia date. Group A1 (n=5), and B1 (n=5) assessed after three days, A2 (n=5), and B2 (n=5) assessed after seven days. 

The animals were kept in the Surgical Technique and Experimental Surgery Laboratory, Surgical Department of Health Science Sector, at UFPR, in a light-dark cycle of 12 hours, temperature (20 ± 2°C), and uncontrolled relative humidity. The animals received commercial food standards for the species and water ad libitum. They were kept in a propylene box, five in each box, with daily hygiene and changes. 

Anesthesia and analgesia were conducted by a veterinarian physician. The anesthesia protocol was initiated with a pre-anesthetic intramuscular injection of ketamine hydrochloride 50mg/kg combined with xylazine hydrochloride 2mg/kg. Anesthetic induction was performed by inhalation with 1% isoflurane, and maintenance with the same drug at 1.5% under mask associated with 100% oxygen. The analgesia was performed via intramuscular injection of tramadol hydrochloride 5mg/Kg in the immediate post-operative period and eight hours after the intervention. 

**Figure 1:**
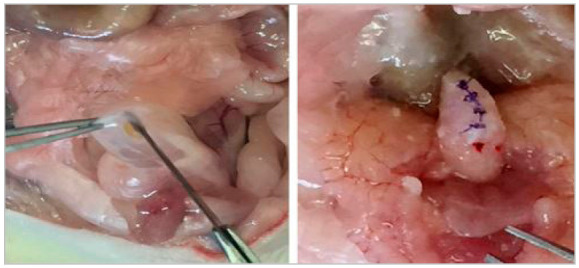
Making the bladder lesion on the left and the complete synthesis of the organ on the right.

After the anesthesia, the animals were identified and had a ventral trichotomy. Then, they were positioned on a surgical board and the antisepsis with a povidone-iodine solution was done. The surgical procedure started by a 5cm median laparotomy and then the urinary bladder was identified. A 0.5cm lesion in the bottom of the bladder was performed. With a surgical magnifying glass, the organ was synthesized in four points using polyglactin 910 6-0 (Figure 1). Laparorrhaphy was sutured in two planes, at first the peritoneum-muscle-aponeurotic plane, with four stitches of continuous running polyglactin 910 sutures, and then skin, with four stitches of continuous running nylon monofilament sutures. 

After recovery from anesthesia, the animals were put back in their original boxes until the expected date for the evaluation.

Euthanasia was performed by a veterinarian physician, according to the protocol described in the CONCEA Euthanasia Practice Guidelines, Resolution nº 37 of the Ministry of Science, Technology, Innovation, and Communication (Brasilia/DF), on February 15, 2018. 

A macroscopic evaluation occurred after the relaparotomy. Bladder integrity, the presence of fluids in the abdominal cavity, as well as the presence of adhesions on the suture line, and urine aspects were observed. Then, the bladder was resected, extended on filter paper, and fixed in 10% formalin for a Pathologist evaluation. 

The paraffin-embedded material was blocked and underwent 5 µm thick sections which were assembled on slides and stained with hematoxylin-eosin technic (HE), to evaluate the wound healing process. Each slide was performed the pathological analysis by reading five fields. For the general analysis, the Vizzotto Junior^17^ et al. method was used. The criteria observed were quality of inflammatory reaction, the intensity of inflammatory reaction, reepithelialization, and angiogenesis, studied on the third and seventh days. The data were classified as severe, moderate, mild, or absent, and transformed into quantitative variables by assignment of indexes to the histological findings, as follows: absent, discrete, moderate, and severe.

Collagen III and collagen I analysis were added by immunohistochemistry and picrosirius-red F3BA staining.

The tissue array or microarray technique was used for immunohistochemical evaluations with the streptavidin-biotin-peroxidase method. Briefly, the cuts were deparaffinized, hydrated, and dipped in phosphate buffer. After antigenic recovery with citrate buffer and endogenous peroxidase block with 0.3% hydrogen peroxide, monoclonal anti-collagen I, anti-collagen III, anti-CD34, and anti-α-smooth muscle actin (anti-SMA) antibodies were applied to each of the slides and incubated overnight. Then the cuts were rinsed in phosphate buffer and a biotinylated secondary antibody was added and incubated for 60 minutes. After a new rinse, the streptavidin-peroxidase complex was applied and the immunohistochemical reaction was revealed with the application of diaminobenzidine. Next, the hematoxylin was counterstaining the cuts, after dehydrating and diaphanized. 

For the analyses, 10 fields from each histological section were read over the scar line and an average was obtained.

Histological slides were stained with Picrosirius-red F3BA and photographed, where each image was captured under normal light and polarized light. Ten fields per wound were selected for the slide readings in an optical microscope with 40x magnification focalized in the wound line. In the picrosirius-red’s analysis, the thicker and birefringent collagen fibers are orange-red (collagen I), and the thinnest and dispersed fibers, weakly birefringent, have greenish color (collagen III). Micrographs were captured by a Sony camera, CCD101. Image analysis was performed using media Cybernetics’ Image-Plus^®^ 4.5 for Windows^®^ application. 

The area of each field read was 142.901 square micrometers. In each one, the percentage of the area occupied by the red and yellow (collagen I) and green (collagen III) fibers was calculated. Considering that the other types of collagens constitute very small fractions, for practical purposes just collagens I and III were considered as the total collagen of the scar. Of the ten fields read, an average was obtained, considered for each animal. 

The reading of sedtions treated by immunohistochemistry to recognize collagens I and III was performed by two pathologists using the qualitative methgod, classifying them as: absent, minimal, moderate and intense.

Analysis of neovascularization was performed by reading ten random fields from the histological sections of the bladder treated with anti-CD34 and anti-SMA, in an area of 131,307.264 µm2 with 10X magnification, scanned with the Axio Scan.Z1 Digital Slide Scanner^®^ (Zeiss, Germany). Virtual slides were evaluated using the Zeiss ZenLite^®^ software (Zeiss, Germany). Vessels labeled with both anti-CD34 and anti-SMA were counted. From the reading of the ten fields, the average was obtained for each animal. The count was performed with the anti-CD34 labeling and them with the anti-SMA in order to have greater security of the count, that is, counter-proof. 

Statistical analysis was descriptive using graphs and tables. The nonparametric test used was Fisher’s test for 2x2 tables, Mann-Whitney, and Student’s t-tests, depending on how the data is described in Gaussian curves or not. The level of rejection for the null hypothesis was p<0.05.

## RESULTS

Throughout the experiment, there were no deaths. 

**Figure 2:**
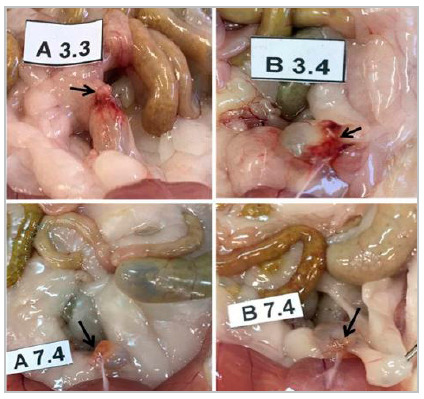
Bladder synthesis line adhesions observed on the third and seventh days. A 3.3: animal nº 3 from group A with 3 days old. B 3.4: animal nº 4 from group B with 3 days old. A 7.4: animal nº 4 from group A with 7 days old. B 7.4: animal nº 4 from group B with 7 days.

On the third and seventh day, in both groups the urinary bladder was intact, there was not fluid in the abdominal cavity and the urine was clean and bloodless. Adhesions were seen in both times evaluated in both groups (Figure 2). 

Mucosal examination showed that it was totally epithelialized on the seventh day, in all urinary bladders, of all animals of this study. 

**Figure 3:**
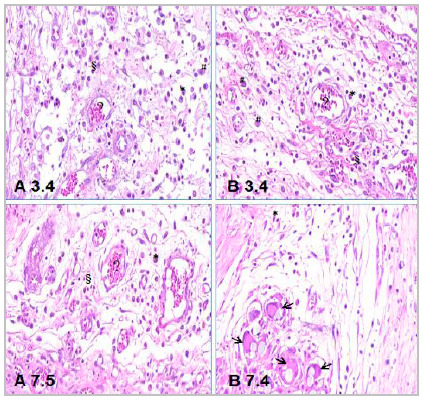
Photomicrographs of histological sections of the bladder of animals from both groups, demonstrating an inflammatory reaction - HE, 200X. A 3.4: animal nº 4 from group A with 3 days old. B 3.4: animal nº 4 from group B with 3 days old. A 7.5: animal nº 5 from group A with 7 days old. B 7.4: animal nº 4 from group B with 7 days old. §Inflammatory infiltrate; #macrophages; *plasma cells; ?vascular congestion; ?foreign body giant cells containing thread fragments.

The inflammatory reaction, evaluated by the method of Vizzotto Junior et al., on the third day was minimal (Fisher’s exact test, p=1.000) and acute (Fisher’s exact test, p=1.000) in both groups. On the seventh day, it was subacute in both groups (Fisher’s exact test, p=1.000) with moderate intensity in the experimental group and minimal in the control group (Fisher’s exact test, p=0,0476) (Figure 3).

The collagen III intensity, marked by immunohistochemistry, was similar in both groups, in the two times studied, was absent or minimal on the third day (p=1.000) and minimal or moderate on seventh day (p=0,2222). Collagen I was absent on the third day in both groups, and but on the seventh day, the moderate amount predominated in the experimental group and minimal in the control group (p=0.0476). 

**Figure 4:**
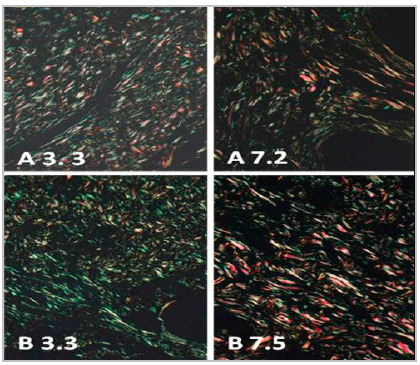
Photomicrographs of histological sections obtained from the third and seventh days of the two groups. Picrosirius red 400X. A 3.3: histological section of the bladder of the animal nº 3, of the third day, of group A. B 3.3: histological section of the bladder of the animal nº 3, of the third day, of group B. A 7.2: histological section of the bladder of the animal nº 2, of the seventh day, of group A. B 7.5: histological section of the bladder of animal nº 5, on the seventh day, in group B. Fibers stained in green = collagen III and those in orange and red = collagen I.

Collagen evaluation by picrosirius-red allowed to verify that the presence of collagen III was similar in both groups (p=0.3312) on the third day, and it was higher in the control group on the seventh day (p=0.0015) (Table 1). Collagen I showed similarity on the third day (p=0.3100) and was higher in the control group on the seventh day (p=0.0015) (Table 2 and Figure 4).

**Table 1:** Average percentage occupied by collagen III, using Picrosirius-red F3BA.

Collagen III area at three days				Collagen III area at seven days		
	Group			Group		
Animal	A	B		Animal	A	B
1	63.95	62.91		1	50.28	65.62
2	75.63	53.00		2	38.10	68.12
3	60.36	61.45		3	39.95	72.35
4	55.77	42.11		4	51.09	79.29
5	49.37	70.74		5	64.66	70.01
Mean	61.02	58.04		Mean	48.82	80.01
SD	9.82	10.91		SD	10.63	5.21
% SD	15.09	19.28		% SD	21.77	9.60

t student test. 3rd day p=0.3312. 7th day p=0.0015. SD: Standard deviation.

**Table 2:** Porcentagem média ocupada por colágeno I, utilizando Picrosirius-red F3BA.

Collagen I area at three days				Collagen I area at seven days		
	Group			Group		
Animal	A	B		Animal	A	B
1	36.05	37.09		1	34.38	49.72
2	24.38	47.00		2	31.87	61.90
3	39.64	38.55		3	27.65	60.05
4	44.22	57.89		4	20.71	48.91
5	50.63	29.26		5	29.99	35.34
Mean	38.98	41.96		Mean	28.92	51.18
SD	9.82	12.40		SD	5.21	10.63
%SD	20.13	28.96		%SD	18.15	20.77

t studant test. 3rd day p=0.3100. 7th day p=0.0015. SD: Standard deviation.

**Figure 5:**
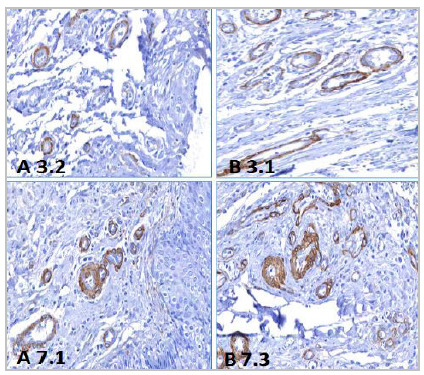
Photomicrographs of histological sections obtained from the third and seventh days of the two groups. Immunohistochemistry, anti-SMA 200X. A 3.2: histological section of the bladder of the animal nº 2, of the third day, of group A. B 3.1: histological section of the bladder of the animal nº 1, of the third day, of group B. A 7.1: histological section of the bladder of animal nº 1, of the seventh day, of group A. B 7.3: histological section of the bladder of animal nº 3, on the seventh day, in group B.

The intensity of angiogenesis assessed by counting vessels marked by anti-SMA showed fewer vessels on the third day (0,0034) and on the seventh day (p=0,0087), both in the group treated with VPA (Table 3 and Figure 5).

**Table 3:** Average of vessels counted in 10 fields for each animal, marked by anti-SMA and the average of the two groups, in the two studied times.

Grupo				
Animal	A3	B3	A7	B7
1	10.4	14.5	12.7	17.2
2	11.4	13.6	15.9	22.7
3	11.1	14.9	16.5	20.9
4	12.5	14.7	17.5	18.0
5	10.4	11.7	15.9	18.9
Mean	11.16	13.88	15.7	19.54
SD	0.87	1.32	1.80	2.24
%SD	7.79	9.51	11.46	11.46
Maximun	12.50	14.90	17.50	22.70
Minimun	10.40	11.70	12.70	17.20

t student test. 3rd day p=0,0034. 7th day p=0,0087.

The counting of vessels marked by anti-CD34, in ten fields, confirmed a lower number of vessels in the treated group, both on the third day (p=0,0006) and on the seventh day (p=0,0072) (Table 4).

**Table 4:** Average of vessels counted in 10 fields for each animal, marked by anti-CD34 and the average of the two groups, in the two studied times.

	Grupo			
Animal	A3	B3	A7	B7
1	11.1	16	15.9	17.1
2	13.2	14.4	15.6	19.9
3	10.2	13.5	18	18.3
4	12	14.7	16.4	20.4
5	11.2	14.9	17.6	19.8
Mean	11.54	14.7	16.7	19.1
SD	1.13	0.90	1.05	1.37
%SD	9.76	6.14	6.31	7.15
Maximun	13.2	16	18	20.4
Minimun	10.2	13.5	15.6	17.1

t student test. 3rd day p=0,0006. 7th day p=0,0072.

## DISCUSSION

Sodium valproate is converted into valproic acid after ingestion and ionized into the gastrointestinal tract to its active form. Its metabolism comprises three oxidation pathways in humans, gluconidation, mitochondrial ß-oxidation, and enzymes of the p450 family (CYP2C9, CYP2A6, and CYP2B6)^7^, which occur in the hepatic level and its administration may generate hepatotoxicity. The pharmacodynamics of the action mechanism comprises increased levels of γ-aminobutyric acid (GABA) in the nervous system (NS), blockade of ionic voltage-dependent channels in the NS, and class I inhibition of the HDACs group^18^. 

Valproic acid was chosen as an experimental drug due to the inhibition potential of several HDACs, especially HDACs 1, 2, and 4. These are divided into 4 classes, with specific intracellular signaling actions and different biochemical patterns. Class I includes HDACs 1,2,3 and 8; in class IIa there are HDACs 4,5,7 and 9; HDAC 6 and 10 are in class IIb and HDAC 11 is in class IV. In the urinary bladder, the HDACs of classes I and IIa are the ones with the highest activity^19^. 

HDACS 1 and 2 are found mainly in the cell nucleus. They are essential for the transcription of proteins such as SIN3A, NuRD, and CoREST, linked to DNA promoters, which produce specific genetic silencing^19^. In addition, this class is essential for cell proliferation and survival by repressing inhibiting proteins of the CDK complex, such as p21WAF1/CIP1 and p57KIP2. Thus, it regulates the transition from phase G1 to phase S of the cell cycle^16^. As class IIa, represented by HDACs 4, has its activity restricted to the local tissue in which it is inserted, it requires joint interaction with class I, because the substitution of tyrosine in the catalytic site of class IIa reduces its enzymatic action^19^.

Wound healing includes wnt^20^ pathways, extracellular signal regulatory kinase (ERK)^21^, and phosphatidylinusitol-3-kinase (PI3-Kinase-Akt-mTOR)^22^, all pathways with frequent errors in human neoplasms^23^. Wnt proved to be a regulator of organogenesis and basal homeostasis, while ERK and PI3-Kinase/Akt are involved in the epithelialization process^23^. With the therapeutic administration of APV, both signaling pathways were altered, thus revealing a possible target for future treatment. In the phase of cell proliferation, VPA interacts with the ß-catenin pathway, molecules that, at high concentrations, induce the increase of mitoses, the regulation of the mesenchymal cell multiplication, motility, and cellular hyperplasia of the scars^23^. In addition, ß-catenin interacts in the transformation of tumor necrosis factor-ß1 (TNF-ß1), increasing its levels that participate in the induction of myofibroblasts differentiation^23^. The increase in ß-catenin levels occurs by the direct interaction of VPA in the Ras protein, showing a viable target in healing^24^.

Furthermore, HDACs inhibitors can decrease hypoxia^25^ and vascular endothelial growth factor (VEGF), inducing angiogenesis^26^ ^-^ ^27^. The formation of new vessels comprises endothelial proliferation, migration, and organization of structural networks forming the vascular tube, both phases modulated by generating nitric oxide (NO)^28^. In vitro, VPA has been shown to inhibit endothelial nitric oxide synthase protein (eNOS), thus directly decreasing NO levels^29^ ^-^ ^30^.

With the reduction of NO synthesis, by decreasing plasma guanylate-mono phosphate cyclase (cGMP) by VPA and the reduction of eNOS, VEGF remains low, thus angiogenesis is compromised^29^. Valproate interacts directly inhibiting angiogenesis in endothelial cells, reducing the rate of mitoses of these cells and their growth^33^. It is also known that the decrease in angiogenesis in urothelium may be caused by the action of VPA increasing the expression of thrombospondin-1 (TSP1)^34^. 

TSP1 is a natural inhibitor of angiogenesis present in tumor cells, such as in bladder cancer. The decrease in TSP expression is directly linked with the ability to induce new vessels, which is necessary for the clonal expansion of cancer cells and metastasis. Moreover, there is an interaction between TSP1 with the expression of VEGF, a molecule responsible for endothelial growth^35^. The antiangiogenic action of TSP1 is mediated at the CD^36^ receptor on endothelial cells, thus initiating an apoptosis cascade^36^. The continuation of this line of research is focused on recognizing the effects of angiogenesis.

The presence of collagen III, which is more fibrillar, when analyzed by the immunolabeling, was minimal on day 3 in both groups and remained similar on day 7 (p=0.2222). When analyzed by picrosirius red under polarized light, there was confirmation for the third day (p=0.3312), but on the seventh day, a higher density was observed in the control group (p=0.0015).

The synthesis of collagen I, when analyzed by immunolabeling, in this study indicated a favorable outcome at seven days of the experiment, in the group treated with valproic acid (p=0.0476). However, this value was very close to the limit for rejection of the null hypothesis, and since the sample size was small, it cannot be stated with certainty. In topical application, in vivo, Lee et al. observed increased levels of α-smooth muscle actin (α-SMA), collagen I and III in the wound, decreasing the distance between scar edges more rapidly^30^. The increase in collagen was also observed by Huang et al. studying the interaction of VPA and atherosclerotic plaques^37^. To have a better perception of the results, the use of picrosirius red staining and examination under polarized light was chosen. On the third day, there was collagen I in small quantity in both groups (p=0.3100). However, it started to exist in greater density in the control group on the seventh day (p=0.0015). 

In cell culture, the reduction in collagen deposition can be explained by the suppression of the cell adhesion molecules α and β integrins, which are required for cell adhesion and deposition. This is how VPA acts in reducing metastasis in bladder cancer, by down-regulating cell surface proteins, as well as decreasing adhesion proteins, even decreasing cell motility; it can also be used as an adjuvant in oncological therapies^14^. 

Furthermore, in culture, Humphrey et al. observed a reduction in collagen after treatment with VPA, showing a decrease in pro-collagen I after 24 hours of immersion^38^. This was also seen by Fuller et al. with dermal fibroblast culture, with a 28% reduction compared to control after 24 hours^39^. The same collagen reduction was observed in the studies by Rishikof et al. by the decrease in mRNA synthesis in lung fibroblasts with the 24-hour treatment^40^. 

Studying bladder culture with VPA, Hodges et al. demonstrated decreased collagen gene expression in muscle cells with reduced mRNA expression of type I and type III collagen^41^. Also, the studies by Rombouts et al. and Mann et al. demonstrated the anti-fibrogenic potential of valproate by hyperacetylation of histones 3 and 4^42^ ^-^ ^43^. 

Thinking about cell migration, the administration of VPA generates a decrease in cell adhesion, especially of tumor cells, as well as modification of cell surface integrins, proteins that are necessary for metastatic invasion of the tumor^20^. In bladder cancer, there was a significant reduction in cell proliferation between G0 and G1, a decrease in the activation of the Akt-mTOR pathway, and inhibition of cell growth^16^. Moreover, there was the confirmation of a greater inflammatory reaction with the use of the drug, by the action of VPA altering the expression of p21 protein, an increase of p27 protein, reduction of cell cycle regulators such as cyclin B and CDK1, besides interleukin 6 (IL-6) and TNF44-45. It is worthwhile mentioning that, in this study, the analysis of the inflammatory reaction was similar in both groups on the third day. However, on day 7, it was moderate in the experimental group, and minimal in the control group (p=0.0476). This level of significance is very close to the adopted limit, and the small sample size does not allow us to state with certainty that there is greater intensity in the experimental group. In both groups, it was acute on the third day and subacute on the seventh day. 

Gene silencing has also been demonstrated after VPA treatment, by methylation of the cytosine-phosphate-guanine nucleotide (CpG) islands present in the promoter site of protein-coding genes, inactivating these cells. Moreover, these changes can be inherited after DNA replication, altering the chromatin structure of the new copies, maintaining the silencing pattern, as shown in certain types of cancers and fibrosis43, besides the possibility of restoring the correct function of genes when treating cases of liver fibrosis^42^. In this study, the analysis of mucosal re-epithelialization showed no significant differences in the two times studied.

There were fewer vessels in the group treated with VPA. This effect, according to Michaelis et al. may be attributed to the direct activity of VPA on endothelial cells. These authors observed inhibition of proliferation, migration, and tube formation of endothelial cells^33^. Engl et al. observed in vivo VPA inhibited angiogenesis. They observed this condition using chicken chorioallantoic membrane assay and in mouse matrigel buffer^46^.

For Cinati et al. the inhibition of angiogenesis could be indirect because treatment of neuroblastoma cells with 1mM valproic acid caused increased expression of antiangiogenic molecules thrombospondin-1 and activin A^47^. In addition, Zgouras et al. showed treatment of the colon adenocarcinoma cell line Caco-2 with VPA caused significant reduction in vascular endothelial growth factor (VEGF) secretion, as well as negative regulation of VEGF mRNA and protein expression^48^.

If we consider that the synthesis of the interstitial matrix and fibrous proteins such as collagens is dependent on oxygen supply, this would explain the lower collagen density in the bladders of the animals in the VPA-treated group.

In a summary analysis, the inflammatory reaction was very similar in both groups. The marking of collagen by the immunolabel showed a small amount, the highest in the experiment group of collagen type I on the seventh day, in qualitative analysis. However, the picrosirius red analysis, of quantitative bias, allowed the recognition of a higher density of collagen type III and collagen type I, on the seventh day, in the control group. It should be considered that an increase in sample size may give greater confidence to the results. It is still important to analyze the interference of VPA on angiogenesis, which has been done in parallel to this study by another researcher from the same study group. 

However, even though the VPA can decrease the intensity of the healing process, it must be admitted that it does not do so in such a way as to lead to risks. It was not observed dehiscence of the synthesis of the bladder, an organ that was intact and protected by adhesions, besides the urine that was clear and of normal aspect. This allows us to think that, when the VPA is used to aid in the treatment of neoplasia and surgical intervention is necessary, it can be done safely.

## CONCLUSION

VPA determined alterations in the bladder healing process, in rats, with lower collagen density and less angiogenic activity, but without compromising the integrity of the organ.
